# Extremely low dose norepinephrine in treatment of refractory chronic cluster headache: two case reports and literature review

**DOI:** 10.3389/fnins.2026.1800150

**Published:** 2026-04-10

**Authors:** Rong Zhou, Cheng Wen, Rulin Geng, Jiawei Liu, Xiaoshuang Xu, Kaixun Huang, Danyu Lin, Enxiang Tao

**Affiliations:** Department of Neurology, The Eighth Affiliated Hospital of Sun Yat-sen University, Shenzhen, China

**Keywords:** chronic refractory cluster headache, norepinephrine, pain, pharmacological management, vasodilation

## Abstract

**Background:**

Refractory chronic cluster headache (rCCH) is a severe form of trigeminal autonomic primary headache that often resists conventional pharmacological treatment, resulting in significant impairment in patients’ quality of life. Identifying effective strategies to reduce attack frequency and shorten cluster periods remains a crucial clinical challenge.

**Case presentation:**

Two cases of rCCH who had suffered from recurrent paroxysmal headaches for several decades were reported. Both had received multiple medications over the long term, with suboptimal response. Given that vasodilation is a key pathophysiological feature of rCCH, an extremely low dose norepinephrine was administered to two cases during acute phase of headache depending on the vasoconstrictive effect of norepinephrine. Their headaches were alleviated significantly through systematic and adjustable titration therapy of extremely low dose norepinephrine for 1–2 weeks. Following treatment, improvements were observed across multiple headache-related scales, including Cluster Headache Impact Questionnaire, Migraine-Specific Quality of Life Questionnaire, Migraine Disability Assessment Questionnaire, Health-Related Quality of Life, Hamilton Rating Scale for Depression and Hamilton Anxiety Scale. After the acute phase, both patients received preventive medications. At one-year follow-up, both patients remained attack-free under regular preventive therapy, had returned to normal daily activities, and maintained baseline scores on headache-related scales.

**Conclusion:**

The administration of norepinephrine in these two cases suggests that extremely low dose norepinephrine may be a potential adjunctive option for acute exacerbations in rCCH patients who are unresponsive to multiple medications. However, further studies are needed to confirm its efficacy and safety.

## 1 Introduction

Cluster headache (CH) is recognized as the most severe form of trigeminal autonomic primary headache, and is classified into episodic cluster headache (ECH), chronic cluster headache (CCH) and probable cluster headache (Membrilla et al., 2025). However, current pharmacological management primarily targets the episodic form (Petersen et al., 2024; Peng and Burish, 2023). Although conventional periodic treatment can alleviate symptoms in some patients, a subset of patients may still progress to refractory chronic cluster headache (rCCH), which complicates therapeutic efforts and potentially leads to disability, significantly impairing patients’ quality of life (Mitsikostas et al., 2014). Thus, identifying strategies to reduce frequency of rCCH and shorten cluster period remains a critical clinical challenge.

Recent studies suggest that vasodilation is a key pathophysiological feature of CH, primarily mediated through trigeminal autonomic vascular reflex (Vila-Pueyo et al., 2025). Activation of this reflex triggers the secretion of neuropeptides, including PACAP-38, acetylcholine, and vasoactive intestinal peptide, which induces vasodilation and sensitizes sensory nerve endings (Frederiksen et al., 2020). Therefore, targeting mechanisms that contribute to vasodilation may help interrupt the cycle of cluster headache attacks. Given that norepinephrine exerts vasoconstrictive effects, this article presents two cases of rCCH treated with extremely low dose norepinephrine during acute exacerbations, aiming to provide clinical insights into the management of CH.

## 2 Case description

### 2.1 Disease procedure

#### 2.1.1 Case 1

A 43-year-old man presented with a 30-year history of recurrent paroxysmal headaches. His headache was characterized by bilateral periocular pressure and soreness. During most attacks, the pain was predominantly on the right side, accompanied by significant anxiety manifesting as restlessness, but without other autonomic symptoms or neurological manifestations. Initially, his headache attacks occurred primarily at night, without any identifiable triggers or prodromal symptoms, and were characterized by abrupt onset and cessation. Headache periods lasted from several days to 2 weeks, with individual attacks lasting 15–30 min. Although medication could terminate acute attacks, it consistently left the patient in pain-free but exhausted state. Over the past 4 years, the patient experienced more frequent cluster headache, lasting 2–3 h daily for 4–5 months each year. In the last 2 years, the headache pattern further evolved to near-daily attacks, occurring 5–8 times per day, with each single attack extending to 1–3 h. His response to previously effective medications diminished, resulting in significantly reduced quality of life.

He has been taking saridon for a long time, with each tablet containing 250 mg acetaminophen, 150 mg isopropylantipyrine, and 50 mg anhydrous caffeine. The dosage of saridon has been gradually increasing, from the initial 1–2 tablets per day to 7–8 tablets per day. Currently, he requires more than 20 tablets daily, yet his headache has not been significantly relieved. Multiple other therapies had also proven ineffective, including sufficient durations of pregabalin (150 mg bid for 3–4 months), calcitonin gene-related peptide monoclonal antibody (galcanezumab, 300 mg/month for 2 months), calcitonin gene-related peptide receptor antagonists (rimegepant, 75 mg qod for 2–3 months), triptans (sumatriptan oral/nasal, used intermittently for acute attacks), flunarizine (10 mg/day for 4 months), and traditional Chinese medicine. He denied smoking, alcohol abuse, or exposure to drugs or toxins. On physical examination, his vital signs and neurological examination were unremarkable.

#### 2.1.2 Case 2

A 67-year-old woman presented with a history of recurrent headaches lasting over 10 years. Her headache attacks were characterized by severe, stabbing pain in the left retro-orbital and peri-orbital regions, radiating to the ipsilateral face and accompanied by ipsilateral eye swelling, tearing, and a facial burning sensation. Headaches were triggered by enclosed environments and sunlight exposure. The headaches exhibited a striking seasonal pattern, recurring in spring or autumn, with each headache period typically lasting 3–4 months. Over the past 3 years, attacks had become nearly daily, occurring 4–6 times per day. Each individual attack lasted 15–60 min and demonstrated remarkable temporal regularity, often occurring 1–2 h after sleep onset.

Her headache was relieved only with analgesic medication. Preventive medications including topiramate (75 mg/day for 5 months), gabapentin (900 mg/day for 3 months) and lithium carbonate (600 mg/day for 3 months, serum level 0.6–0.8 mEq/L) were ineffective in reducing her headache attacks. On examination, she was very restive, although her vital signs and neurological examination showed no significant abnormalities.

### 2.2 Auxiliary examination

Laboratory tests revealed mild abnormalities. In Case 1, blood glucose (6.45 mmol/L; normal range 3.9–6.2 mmol/L) and HbA1c (6.9%; normal range 4–6%) were mildly elevated, while other biochemical parameters were normal. In Case 2, mild elevations were noted in serum total cholesterol (5.72 mmol/L; normal range 2.83–5.20 mmol/L), triglycerides (1.99 mmol/L; normal range 0.45–1.70 mmol/L), uric acid (362.09 μmol/L; normal range 89–357 μmol/L), and rheumatoid factor (69.2 IU/mL; normal range 0–20 IU/mL).

Prior to treatment, both patients underwent comprehensive cardiovascular evaluation including: (1) detailed medical history focusing on cardiovascular disease, hypertension, diabetes, smoking and drinking alcohol; (2) physical examination including cardiac auscultation and peripheral pulse assessment; (3) laboratory testing including cardiac enzymes; (4) 12-lead electrocardiogram; (5) transthoracic echocardiogram; (6) contrast-enhanced transcranial Doppler with bubble study. The above examinations showed no significant abnormalities.

**Figure 1:**
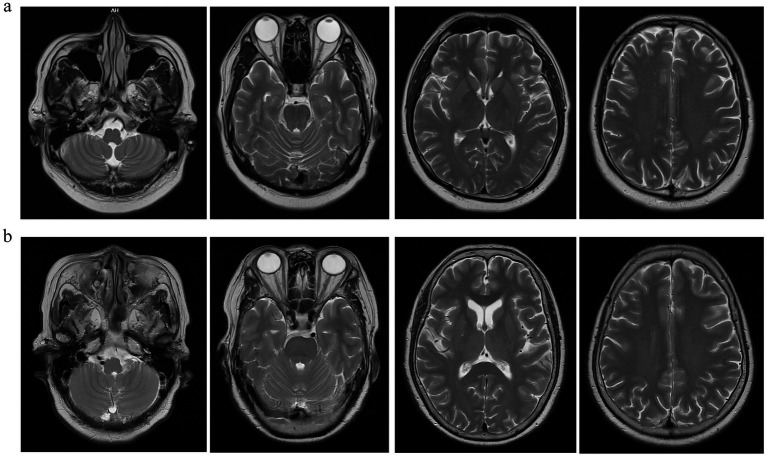
Brain MRI examination images of two cases. **(a)** Brain MRI images of Case 1. **(b)** Brain MRI images of Case 2.

What’s more, their electroencephalogram showed no significant abnormalities. Brain MRI of Case 1 showed no obvious structural lesions (Figure 1a). Brain MRI of Case 2 demonstrated mild non-specific white matter lesions (Figure 1b).

### 2.3 Diagnosis and treatment

Based on their clinical presentations and ancillary test results, both patients were diagnosed with acutely exacerbated refractory chronic cluster headache according to the International Classification of Headache Disorders (3rd edition) (International Headache Society, 2018) (Table 1) and European Headache Federation (EHF) consensus definition of refractory chronic cluster headache (Mitsikostas et al., 2014).

**Table 1:** Diagnosing basis of both patients with CH according to ICHD-3 diagnostic criteria.

ICHD-3 diagnostic criteria	Case 1	Case 2
At least 5 attacks fulfilling criteria B–D	A 30-year history of recurrent paroxysmal headaches The frequency of headache attacks has increased year by year	A history of recurrent headaches lasting over 10 years Over the past three years, the attacks have become almost daily, with more than 5 attacks
Severe or very severe unilateral orbital, supraorbital, and/or temporal pain lasting 15–180 min (when untreated)	His headache was characterized by bilateral periocular pressure and soreness. During most attacks, the pain was predominantly on the right side The lasting time of individual attacks increased from 15–30 min to 1–3 h	Classic unilateral orbital pain Temporal regularity: individual attack lasted 15–60 min
Either or both of: ipsilateral conjunctival injection and/or lacrimation, a sense of restlessness or agitation	During most attacks, he was significant anxiety manifesting as restlessness, but without other autonomic symptoms	Ipsilateral autonomic symptoms (lacrimation, eye swelling). She was extreme restive
Frequency of attacks every other day to 8 per day	Over the past two years, the headache pattern has further evolved to near-daily attacks, occurring 5–8 times per day	Over the past three years, the attacks had become nearly daily, occurring 4–6 times per day
Not better accounted for by another ICHD-3 diagnosis	None meets another diagnosis criteria of ICHD-3	None meets another diagnosis criteria of ICHD-3

Given that both patients had previously received multiple preventive and acute treatments, especially Case 1, who had previously taken high doses of saridon, medication overuse headache (MOH) was considered in the differential diagnosis. However, the diagnosis of MOH requires that headaches worsen during medication overuse and improve after withdrawal. In Case 1, the headache frequency and severity did not improve after stopping saridon, which argues against MOH as the underlying diagnosis. Other differential diagnoses, such as paroxysmal hemicrania or SUNCT syndrome, were considered but ruled out based on the attack duration, frequency, and lack of response to non-steroidal anti-inflammatory drugs.

**Figure 2:**
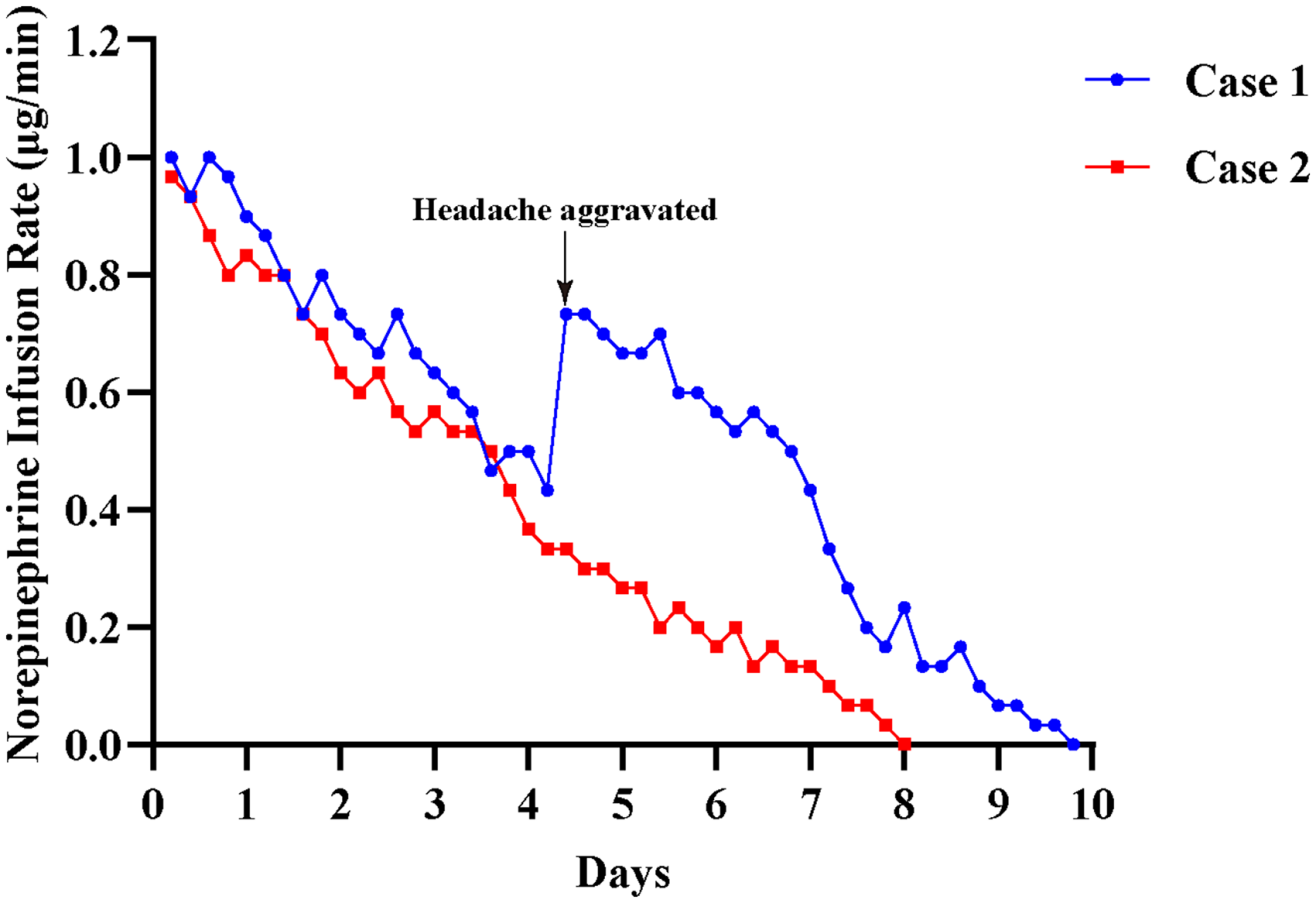
Norepinephrine infusion rates over time in two patients with refractory chronic cluster headache. The line graph depicted the daily norepinephrine infusion rates (μg/min) for Case 1 (blue line) and Case 2 (red line) during hospitalization. Norepinephrine bitartrate (1 mg) was diluted in 500 mL of 0.9% saline solution, yielding a final concentration of 2 μg/mL (based on norepinephrine base equivalent). This extremely dilute solution was administered via a microprocessor-controlled infusion pump to ensure precise and stable delivery rates. The infusion protocol was as follows: Acute attack termination: Initial infusion rate of 20-30 mL/h, corresponding to 0.67-1.0 μg/min of norepinephrine base for acute headache relief. Maintenance phase: Following acute relief, the infusion rate was reduced to 10-15 mL/h for 2-3days, corresponding to 0.33-0.5 μg/min. Tapering phase: Over the subsequent 2-3 days, the rate was gradually reduced to 5 mL/h (0.17 μg/min) and then discontinued.

Upon admission, both patients received comprehensive treatment for 2–3 days according to the aforementioned guidelines, including NSAIDs, tramadol, pregabalin, and oxygen therapy. However, their headaches were not relieved. After excluding contraindications of norepinephrine, including a history of myocardial infarction or unstable angina, heart failure (NYHA class II–IV), uncontrolled hypertension (systolic BP >160 mmHg despite treatment), clinically significant arrhythmias, significant carotid or cerebral artery stenosis, pheochromocytoma, or hypersensitivity to norepinephrine, extremely low dose norepinephrine (1 mg diluted in 500 mL of 0.9% saline) was administered via a micro-infusion pump. For acute headache relief, the infusion rate was titrated between 20–30 mL/h (0.67–1.0 μg/min), while a maintenance infusion rate of 10–15 mL/h (0.33–0.5 μg/min) was used to prevent recurrence. Then the infusion rate was gradually reduced to 5 mL/h (0.17 μg/min) and tapered off over the following 2–3 days. Throughout the treatment, the norepinephrine bitartrate dosage was individually adjusted according to patients’ clinical response (Figure 2).

Continuous electrocardiographic and non-invasive blood pressure monitoring were performed throughout the norepinephrine infusion period, and patients were closely monitored for any potential adverse effects. Vital signs were recorded at baseline, every 15 min during the first hour of infusion, every 30 min for the next 2 h, and hourly thereafter until 2 h after infusion discontinuation. Any hypertensive episode (defined as systolic BP >150 mmHg or diastolic BP >95 mmHg) or arrhythmia was documented, which would prompt immediate dose reduction or cessation. No arrhythmias, palpitations, chest pain, or other cardiovascular adverse events were observed in either patient throughout the treatment period. Specific hemodynamic data for both patients were as follows:

#### 2.3.1 Case 1

- Baseline BP: 132/84 mmHg, HR: 78 bpm.
- During infusion (0.67–1.0 μg/min): BP range 126–135/80–88 mmHg, HR range 74–82 bpm.

One transient BP elevation to 152/94 mmHg occurred during headache aggravated, which resolved without intervention within 10 min, and no dose adjustment was required.

- At maintenance rate (0.33–0.5 μg/min): BP range 122–134/78–86 mmHg, HR range 72–80 bpm.
- During taper (0.17 μg/min): BP returned to baseline levels (128–135/80–84 mmHg).

#### 2.3.2 Case 2

- Baseline BP: 128/76 mmHg, HR: 72 bpm.
- During infusion (0.67–1.0 μg/min): BP range 122–138/70–82 mmHg, HR range 68–76 bpm.
- At maintenance rate (0.33–0.5 μg/min): BP range 118–132/68–80 mmHg, HR range 66–74 bpm.
- During taper (0.17 μg/min): BP and HR remained stable within normal limits.

No hypertensive episodes or significant heart rate variability (> ±10% from baseline) were observed.

Following treatment, headache symptoms were markedly relieved in both cases. At discharge, Case 1 was prescribed methylprednisolone (40 mg, once daily), verapamil hydrochloride tablets (80 mg, three times daily), and topiramate tablets (25 mg, twice daily). Case 2 was prescribed verapamil hydrochloride tablets (80 mg, twice daily) and paroxetine hydrochloride tablets (10 mg, once daily) to prevent the onset of headaches.

Additionally, a series of headache-related scales—including Cluster Headache Impact Questionnaire, Migraine-Specific Quality of Life Questionnaire (MSQ), Migraine Disability Assessment Questionnaire (MIDAS), Health-Related Quality of Life (HR-QOL), Hamilton Rating Scale for Depression (HRSD), and Hamilton Anxiety Scale (HAMA)—were evaluated for both cases before treatment and on day 7 after discharge. Compared with the baseline, the frequency of headache attacks and acute medication use of two cases were decreased, their MSQ score, MIDAS score, HRSD score and HAMA score were also decreased, and scores of different dimensions of HR-QOL scale were increased after treatment (Table 2).

**Table 2:** Headache related scales scores before treatment and after discharge.

Scales		Case 1		Case 2	
		Pre-treatment	Seven days after discharge	Pre-treatment	Seven days after discharge
Cluster Headache Impact Questionnaire	Frequency of attack	8	1	10	4
	Frequency of acute medication use	8	1	8	4
Migraine-Specific Quality Life Questionnaire		48	13	56	24
Migraine Disability Assessment Questionnaire		300	125	450	175
Hamilton Rating Scale for Depression		27	11	35	14
Hamilton Anxiety Scale		24	8	39	15
Health-Related Quality of Life	Physical function	75	84	69	81
	Role physical	57	78	53	72
	Bodily pain	40	83	41	69
	General health	62	80	64	78
	Vitality	48	79	53	71
	Social function	64	76	66	73
	Role emotional	61	82	58	77
	Mental health	56	77	59	70

## 3 Discussion

Cluster headache is one of the most severe primary headache disorders, classified into episodic and chronic forms. Some patients with episodic CH may transition to CCH, and eventually to rCCH (Goadsby, 2024; Mitsikostas et al., 2014). rCCH is associated with an elevated risk of mental disorders, disability and suicidality (Wei and Goadsby, 2021). Unfortunately, effective treatment for rCCH remains challenging (Hoffmann et al., 2019), as illustrated by the two cases presented above, who experienced persistent headaches despite trials of multiple preventive and acute treatments.

The pathophysiology of cluster headache involves in a complex interplay among hypothalamus, trigeminovascular system and autonomic nervous system. Hypothalamic dysfunction is thought to act as the primary “pacemaker” generating the characteristic circadian and circannual periodicity of CH (Islam et al., 2024). Pain in rCCH, typically localized to the periorbital and temporal regions, is believed to be mediated by activation of the trigeminovascular system. When the trigeminal nerve is stimulated, nociceptive nerve fiber endings release vasoactive neuropeptides (including calcitonin gene-related peptide, substance P, neurokinin A, vasoactive intestinal peptide and pituitary adenylate cyclase activating peptide) leading to neurogenic inflammation and vasodilation of dural and pial blood vessels (Bertotti et al., 2025; D’Andrea et al., 2019). Concurrently, parasympathetic outflow from the superior salivatory nucleus via the sphenopalatine ganglion produces the cranial autonomic features of CH (Lund et al., 2023). Moreover, central sensitization within the trigeminocervical complex and thalamocortical pathways contributes to pain chronicity and referral patterns (Tao et al., 2025), and long-term chronic and recurrent episodic headaches may sensitize pain pathways and lower pain thresholds, establishing a vicious cycle that promotes progression to rCCH (Messina et al., 2023). Therefore, interrupting this vicious cycle is crucial in rCCH management, and targeting vasodilation represents a rational therapeutic strategy in rCCH.

In both patients, headache attacks were alleviated following treatment with extremely low dose norepinephrine. Beyond its well-established vasoconstrictive effects, norepinephrine may exert therapeutic effects in rCCH through several additional mechanisms. Firstly, norepinephrine is a key neurotransmitter in the descending pain modulatory system originating from the locus coeruleus and brainstem nuclei. Through activation of α_2_-adrenergic receptors in the spinal dorsal horn and trigeminal nucleus caudalis, it inhibits nociceptive transmission via both pre-synaptic and post-synaptic mechanisms (Pertovaara, 2006). Exogenous administration of low dose norepinephrine may enhance this endogenous pain-inhibitory pathway. Secondly, norepinephrine may help stabilize autonomic nervous system function. CH attacks are characterized by parasympathetic hyperactivity and sympathetic dysfunction. Lower serum norepinephrine levels have been observed in CH patients compared with healthy controls (Mo et al., 2022), suggesting that sympathetic hypofunction may contribute to the pathophysiology of CH. Supplementing norepinephrine could therefore help restore autonomic balance and reduce parasympathetically mediated vasodilation. Thirdly, adrenergic receptors are expressed on trigeminal sensory neurons, and norepinephrine may directly modulate their excitability and neuropeptide release (Donertas-Ayaz and Caudle, 2023), potentially attenuating trigeminal nerve activation.

In the cases presented above, extremely low dose norepinephrine was administered under close monitoring, with no significant adverse effects. One notable observation was transient worsening of headache in Case 1 during treatment, which was attributed to overly rapid reduction of the infusion rate. This observation suggests that gradual tapering of norepinephrine infusion may be important to avoid rebound worsening of headache. Following the acute phase, two patients are administered preventive medications, including verapamil, pregabalin and paroxetine to reduce frequency of CH attacks. Meanwhile, they are also instructed on avoiding known triggers, such as sleep deprivation, fatigue, smoking, and prolonged sun exposure. At one-year follow-up, both patients remained attack-free on regular preventive therapy, had returned to normal daily activities, and maintained improved scores on headache-related scales.

These observations suggest that extremely low dose norepinephrine infusion was well tolerated in these two cases and may represent a potential therapeutic option for acute exacerbations of rCCH. Firstly, the dose administered (0.17–1.0 μg/min) is substantially below the typical pressor range (2–20 μg/min), achieving local cranial vasoconstriction without systemic hemodynamic effects. Secondly, continuous ECG and non-invasive blood pressure monitoring, together with predefined stopping criteria ensured timely detection and management of any potential adverse events. Last but not least, two cases maintained stable hemodynamics throughout treatment, without any serious side effects. The only notable event was a transient, self-limited blood pressure elevation in Case 1 during headache exacerbation, which was resolved without intervention.

However, it is important to acknowledge that the clinical improvement observed in both patients cannot be definitively attributed solely to norepinephrine infusion, given the presence of multiple concomitant treatments and the potential for natural fluctuation in cluster headache periods. For these two patients, the temporal association between norepinephrine infusion and acute improvement is suggestive, as both patients were received extremely low dose norepinephrine after failing multiple other medications. However, due to individual variability and the limitations of case reports, the causality of norepinephrine in acute treatment of CCH remains limited. Therefore, more controlled studies are required to evaluate its efficacy and safety.

## 4 Conclusion

In summary, the management of rCCH remains clinical challenge due to its complex pathophysiology and limited treatment options. Above observations suggest that extremely low dose norepinephrine infusion was well tolerated in these two patients with rCCH and may represent a potential adjunctive option for managing acute exacerbations. However, given the inherent limitations of case reports, including the absence of a control group, concurrent use of multiple medications, and potential for natural disease fluctuation, controlled studies are needed to evaluate its efficacy and safety.

## Data Availability

The original contributions presented in the study are included in the article/supplementary material, further inquiries can be directed to the corresponding authors.
